# Uneven-Layered Coding Metamaterial Tile for Ultra-wideband RCS Reduction and Diffuse Scattering

**DOI:** 10.1038/s41598-018-26386-5

**Published:** 2018-05-25

**Authors:** Jianxun Su, Huan He, Zengrui Li, Yaoqing (Lamar) Yang, Hongcheng Yin, Junhong Wang

**Affiliations:** 1 School of Information Engineering, Communication University of China, Beijing, 100024 China; 2 Department of Electrical and Computer Engineering, University of Nebraska-Lincoln, Lincoln, NE 68182 USA; 3 National Electromagnetic Scattering Laboratory, Beijing, 100854 China; 4 Institute of Lightwave Technology, Beijing Jiaotong University, Beijing, 100854 China

## Abstract

In this paper, a novel uneven-layered coding metamaterial tile is proposed for ultra-wideband radar cross section (RCS) reduction and diffuse scattering. The metamaterial tile is composed of two kinds of square ring unit cells with different layer thickness. The reflection phase difference of 180° (±37°) between two unit cells covers an ultra-wide frequency range. Due to the phase cancellation between two unit cells, the metamaterial tile has the scattering pattern of four strong lobes deviating from normal direction. The metamaterial tile and its 90-degree rotation can be encoded as the ‘0’ and ‘1’ elements to cover an object, and diffuse scattering pattern can be realized by optimizing phase distribution, leading to reductions of the monostatic and bi-static RCSs simultaneously. The metamaterial tile can achieve −10 dB RCS reduction from 6.2 GHz to 25.7 GHz with the ratio bandwidth of 4.15:1 at normal incidence. The measured and simulated results are in good agreement and validate the proposed uneven-layered coding metamaterial tile can greatly expanding the bandwidth for RCS reduction and diffuse scattering.

## Introduction

In recent years, there have been abundant researches on manipulating electromagnetic waves in order to realize the stealth of targets. As a new category of metamaterials, metasurfaces^1,2^ are widely utilized in many fields because of their low profiles and potential abilities of controlling electromagnetic (EM) waves, such as polarization converter^3^, ultra-thin metalenses^4,5^, low scattering^6–8^, wave plates for generating vortex beams^9,10^, and electromagnetic interference and shielding^11^.

To reduce the RCS of a structure, the method of devising the well-known radar absorbing metamaterials (RAMs) to the surface of the objects is introduced. The RAM is capable of transforming electromagnetic energy into heat^12^. It is easy to manufacture and can efficiently suppress the RCS of the targets. However, most of RAMs usually have narrow bandwidth because of operating in the vicinity of resonance frequency^13,14^. Another method is to exploit the cancellation effects arising from the well-known 180°phase-difference between the corresponding reflection coefficients. The basic way is to employ the perfect electric conductor (PEC) and artificial magnetic conductor (AMC) together to design metasurface. The backscattering field can be effectively cancelled by redirecting it along other angles. However, the bandwidth is really limited due to the narrow in-phase reflection bandwidth of the AMC^15^. Then, a planar chessboard-like metasurface is proposed to reduce RCS. Two AMC cells based on Jerusalem Cross configuration^16^ have been used to obtain −10 dB monostatic RCS reduction over 41% frequency bandwidth. The dual electromagnetic band-gap (EBG) surfaces are adopted to obtain −10 dB RCS reduction over 60% frequency bandwidth^17^. Non-absorptive two-layered miniaturized-element frequency selective surfaces of a chessboard-like configuration was proposed for wideband RCS reduction^18^. The −10 dB RCS reduction bandwidth of 50% is achieved at the normal incidence. A planar chessboard structure consisting of saltire arrow and four-E-shaped unit cells is presented in the design^19^. A broad bandwidth of 85% is obtained for −10 dB RCS reduction. A combination of absorptive and phase gradient metasurfaces was presented in^20^, and the −10 dB RCS reduction was realized in dual-band covering 2.7–3.5 GHz (25% bandwidth) in S band and 10.5–18 GHz (52.63% bandwidth) in X and Ku band. Then, the dual wideband checkerboard surfaces are presented^21^, and the −10 dB RCS reduction, in the frequency bands of 3.94–7.40 GHz and 8.41–10.72 GHz is about 61% and 24% bandwidths by utilizing two dual-band EBG structures. Furthermore, the multi-resonance method is also employed in^22^. The metasurface consisting of four subarrays randomly distributed reflection phases at four specific frequencies achieves 52.63% bandwidth for −10 dB RCS reduction. Another method is to utilize the polarization conversion metasurface to realize wideband RCS reduction. An ultra-wideband polarization reflective surface with a periodic array of quasi-L-shaped patches is presented in^23^, which obtains about −5 dB RCS reduction in the frequency band of 6–19 GHz (104% bandwidth). In^24^, the metasurface is composed of square and L-shaped patches, which can convert the polarization of the incident wave to its cross-polarized direction, representing the plasmon cloaking of an object. A −10 dB RCS reduction is achieved over an ultra-wideband of 98%.

Recently, coding or digital metasurface has been proposed for wideband RCS reduction^25–27^. In^25^, the 1-bit, 2-bit and 3-bit coding metasurfaces composed of digital elements have been proposed and obtains −10 dB RCS reduction bandwidth of 66.67%. A 3-bit coding metasurface based on multi-resonant polarization conversion elements is presented in^26^. The bandwidth of −10 dB RCS reduction is 89.9%. A broadband and broad-angle polarization-independent random coding metasurface for RCS reduction is proposed in^27^. The −10 dB RCS reduction bandwidth of 84.75% is realized.

The aim in this paper is to present a novel uneven-layered metamaterial tile for ultra-wideband RCS reduction. Two unit cells with 180° (±37°) phase difference in ultra-wide frequency band are exactly designed to build the metamaterial tile, resulting in ultra-wideband phase cancellation. Metamaterial tile with small size is universal and easy to be modularized in production and processing. Furthermore, it can be encoded as digital element to cover objects and obtain diffuse scattering pattern, leading to low bi-static RCS. The simulated and measured results show that −10 dB RCS reduction is achieved over an ultra-wide frequency band from 6.2 to 25.7 GHz (ratio bandwidth of 4.15:1). The proposed uneven-layered metamaterial tile can greatly expanding the bandwidth for RCS reduction and diffuse scattering and may find potential applications in stealth technology.

## Results

### Unit cell design

**Figure 1:**
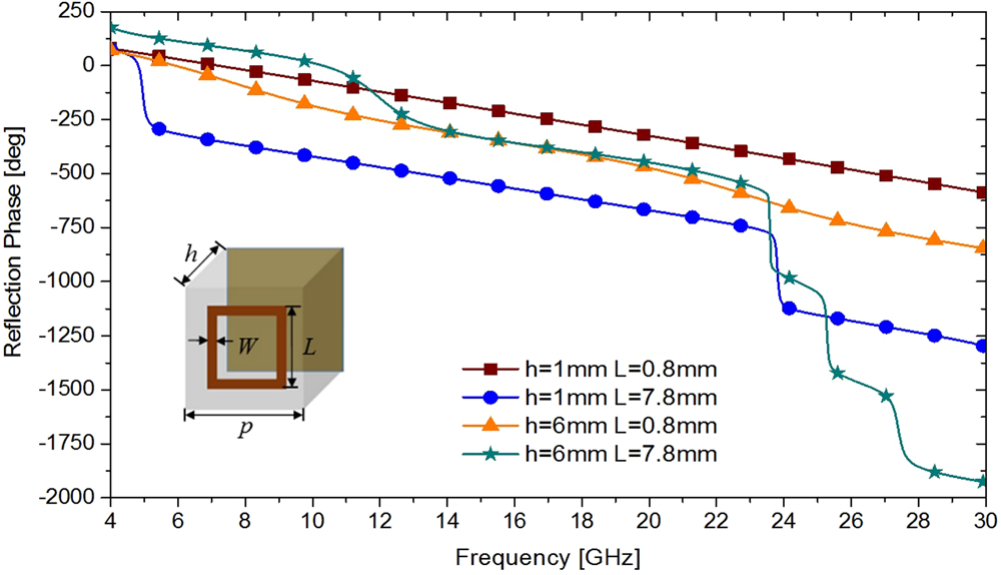
Four reflection phase versus frequency curves for the maximum and minimum values of thickness *h* and side length *L*.

Square ring metallic patch is chosen as the basic shape of the unit cell. The square ring patch has side length of *L* and width of *W* as depicted in the inset of Fig. 1. It is printed on the F4B-2 substrate with thickness of *h* and a relative permittivity of 2.65. The unit cells are simulated by Frequency Domain Solver of CST Microwave Studio with unit cell boundary conditions to achieve the reflection coefficients. In simulation, the side length *L* of unit cell varies from 0.8 to 7.8 mm with a step width of 0.1 mm and the thickness *h* of substrate varies from 1 to 6 mm with a step size of 1 mm. The width *W* of square ring is 0.3 mm and the period *p* of the unit cell is 8 mm. The reflection phase versus frequency for the maximum and minimum values of *L* and *h* is illustrated in Fig. 1. For the unit cell of any size, the reflection phase curves are within the four curves.

The RCS reduction for the metamaterial tile is dependent on the phase difference between two unit cells according to^25^

1 $$RCSreduction=10\,\mathrm{lg}\,|\frac{{e}^{j{\phi }_{1}}+{e}^{j{\phi }_{2}}}{2}{|}^{2}$$

where *φ*_1_ and *φ*_2_ are the reflection phases of the two unit cells. To achieve −10 dB RCS reduction, reflection phase difference between two unit cells must vary from 143° to 217° while the magnitude of the reflection coefficient is unity because the PEC ground plane is infinite. We look for two unit cells with 180° (±37°) phase difference in the largest possible frequency band.

**Figure 2:**
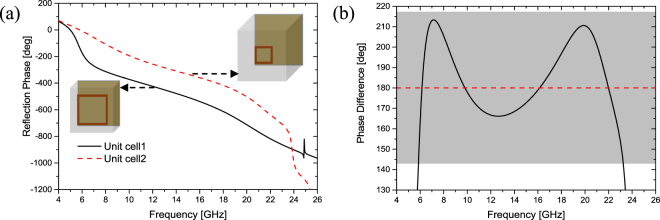
The reflection phases of two unit cells and phase difference between them. (**a**) Reflection phases. (**b**) Phase difference.

Finally, this searching process results in two dimensions. The thickness *h*_1_ and side length *L*_1_ of one unit cell are 3 mm and 7 mm, respectively. The other is with thickness of *h*_2_ = 6 mm and side length of *L*_2_ = 2.6 mm. The reflection phases of two unit cells and the phase difference between them are illustrated in Fig. 2. Apparently, we can obtain 180° (±37°) phase difference from 5.88 GHz to 23.25 GHz (up to a ratio bandwidth of 4:1), and the phase difference is exactly 180° at 6.16 GHz, 9.8 GHz, 16.13 GHz and 22 GHz. Therefore, an ultra-broadband RCS reduction of the proposed metasurface is expected.

### Metamaterial tile design

**Figure 3:**
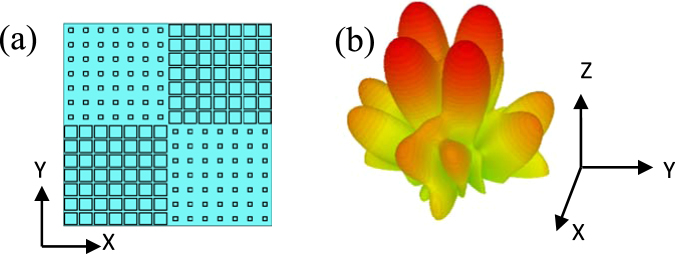
The metamaterial tile and its scattering characteristics. (**a**) The geometry of the metamaterial tile. (**b**) The bi-static scattering pattern.

**Figure 4:**
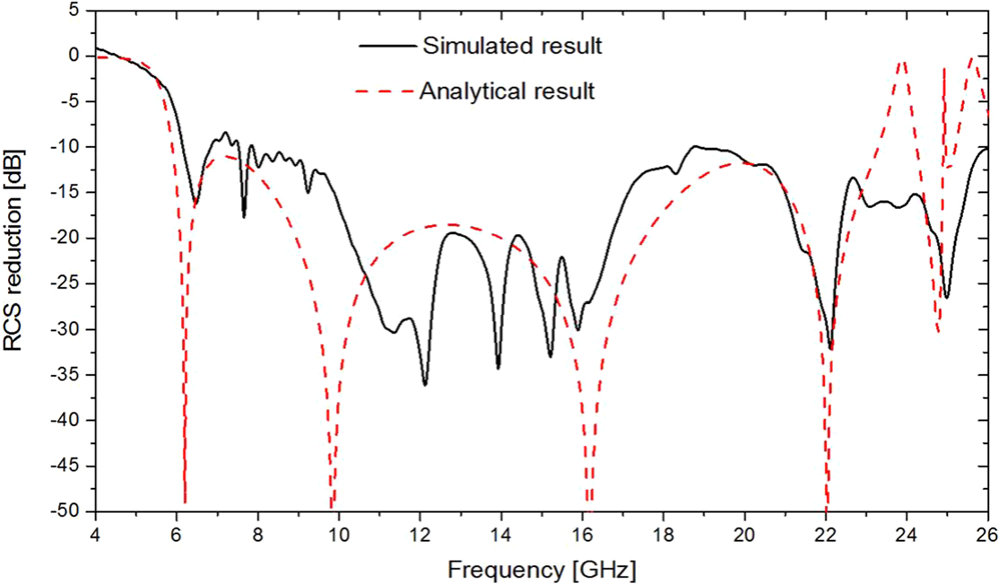
The simulated and analytical RCS reductions of the metamaterial tile for normal incidence.

In order to approximately satisfy the unit cell boundary condition used in simulation, a lattice consists of 7 × 7 unit cells. A metamaterial tile with overall dimension of 112 mm × 112 mm contains 2 × 2 lattices and two different lattices are distributed alternately as depicted in Fig. 3(a). To observe the scattering behavior, the metamaterial tile is simulated by the Transient Solver of CST Microwave Studio. The scattering pattern of the metamaterial tile has four main lobes deviating from the normal direction as shown in Fig. 3(b). An equal-sized PEC ground plane is also simulated as a reference. The RCS of the metamaterial tile and an equal-sized PEC ground plane are separately simulated first. Then, subtraction is made between their values to get the RCS reduction. The analytical and simulated RCS reductions of the metamaterial tile under normal incidence are in good agreement, as shown in Fig. 4. There are some derivations between the simulated and analytical results because Eq. (1) doesn’t consider the coupling effect between neighboring lattices and edge diffraction at the open boundary. The simulated RCS reduction at 6.8 GHz declines slightly. The metamaterial tile can achieve the simulated RCS reduction less than −8.5 dB from 6.1 GHz to 26 GHz with a ratio bandwidth of 4.26: 1.

It should be mentioned that the RCS reduction can be approximated by Eq. (1), which provides a good guideline for RCS reduction of the metamaterial tile compared to that of a PEC ground plane.

### Diffuse scattering of electromagnetic waves

**Figure 5:**
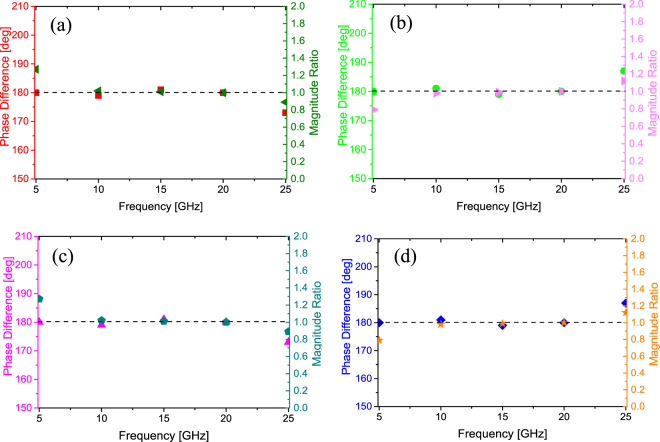
The phase difference and magnitude ratio of electric fields of four main scattering beams between the metamaterial tile and its 90-degree rotation. (**a**) *φ* = 45°. (**b**) *φ* = 135°. (**c**) *φ* = 225°. (**d**) *φ* = 315°.

This section introduces how to use metamaterial tiles as encoding elements to cover an object and achieve diffuse scattering of electromagnetic waves. The metamaterial tile has four scattering beams directing to (*θ*, 45°), (*θ*, 135°), (*θ*, 225°) and (*θ*, 315°). The elevation angle *θ* is calculated by^25^

2 $$\theta =\arcsin (\frac{\lambda }{\sqrt{2}D})$$

where *λ* and *D* are the working wavelength and the length of a lattice, respectively. The values of the angle *θ* at 5 GHz, 10 GHz, 15 GHz, 20 GHz and 25 GHz are 49.25°, 22.26°, 14.63°, 10.92° and 8.72° respectively. Total 20 field probes are set in CST to probe the electric fields in far field for four scattering beams at these five frequencies. Figure 5 shows the simulated phase difference and magnitude ratio of reflection coefficients between the metamaterial tile and its 90-degree rotation. It is noting that the scattering fields of each beam between the metamaterial tile and its 90-degree rotation are approximate equal amplitude and opposite phase. Small deviations are attributed to the theoretical scattering angles obtained by Eq. (2) deviating from that of the simulation, resulting in the field probe not pointing to the beams exactly. Thus, the metamaterial tile and its 90-degree rotation are nominated as “0” and “1” elements, respectively. These two encoding elements can be used to cover an object of any shape and achieve the diffuse scattering through optimizing the phase layout. The metamaterial tile with the small size is easy to be modularized and in mass production for commercial application.

To illustrate the mono- and bi-static scattering characteristics, here we design a metasurface as an example composed of 4 × 4 metamaterial tiles with total dimension of 448 mm × 448 mm. As expected, the bi-static scattering pattern of chessboard-like metamaterials can be analyzed by array theory^28^. For a metasurface consisting of M × N lattices, the lattices are spaced with *d*_x_ in the *x* direction and *d*_y_ in the *y* direction. The scattering pattern of the metasurface at angle *θ* and *φ* is given by

3 $$\sigma (\theta ,\phi )=EP(\theta ,\phi )\cdot AF(\theta ,\phi )$$

where *EP* and *AF* are the element pattern, namely, the scattering pattern of the metamaterial tile, and array factor, respectively. *θ* and *φ* are the elevation and azimuth angles of an arbitrary scattering direction, respectively. In our model, we assume that the *EP* is fixed. According to the array theory, the *AF* can be expressed by

4 $$AF(\theta ,\phi )=\sum _{m=1}^{M}\sum _{n=1}^{N}\exp \{\,-\,j[2\pi \,\sin \,\theta (\cos \,\theta \cdot m{d}_{x}+\,\sin \,\phi \cdot n{d}_{y})/\lambda +\varphi (m,n)]\}$$

**Figure 6:**
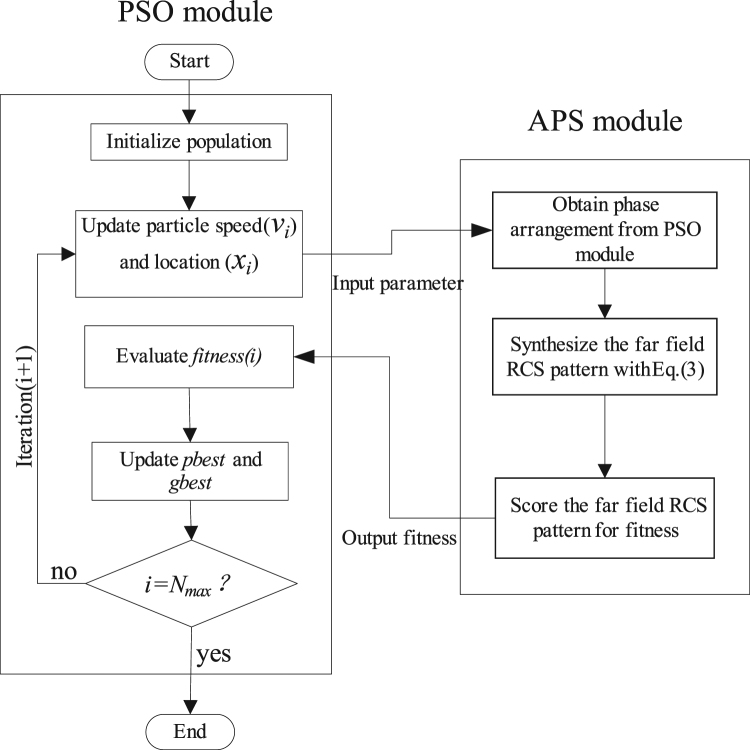
The flowchart for hybrid APS and PSO algorithm.

**Figure 7:**
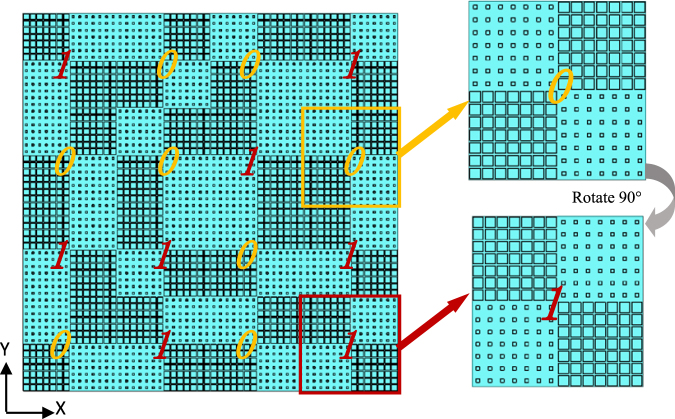
The coding matrix of elements of the metasurface and the details of the two encoding elements: “0” element and “1” element.

*ϕ*(*m*, *n*) is the initial phase of the element, which has two phase values 0 or π to be chosen. To obtain the diffuse scattering pattern, the array theory together with particle swarm optimization (PSO) algorithm is adopted to optimize and find the best phase layout^29^. The flowchart of the hybrid array pattern synthesis (APS) and particle swarm optimization (PSO) method is shown in Fig. 6. The PSO module updates the particle speed and the population location (i.e. the phase arrangements) in each iteration, and then sends the information to the APS module. The latter gives the scattering pattern of the optimized metasurface based on array pattern synthesis and calculates the maximum RCS and monostatic RCS. The fitness is then scored and returned to the PSO module and the PSO module evaluates the fitness. After a number of iterations, we can get the optimal phase arrangement for the metasurface with a lowest RCS as required. The kernel of this flowchart is that the PSO module determines various combinations of basic metamaterial tile, whose performances are judged by the APS module to find thebest solution. Particle swarm optimization is a high-performance optimizer that is very easy to understand, easy to implement and highly robust. It is similar in some ways to genetic algorithms, but requires less computational bookkeeping and generally only a few lines of code. Finally, the coding matrix of elements is obtained as shown in Fig. 7.

**Figure 8:**
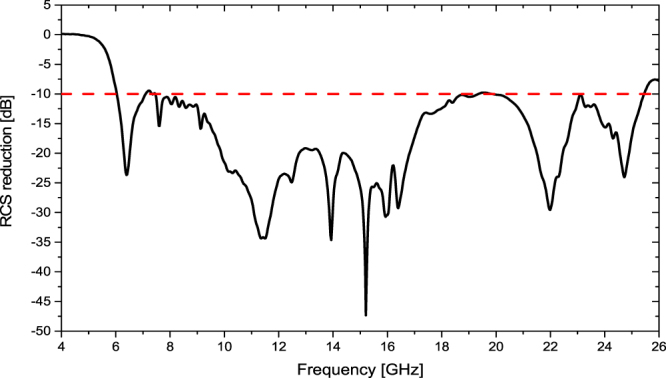
The simulated result of RCS reduction of the metasurface for normal incidence.

**Figure 9:**
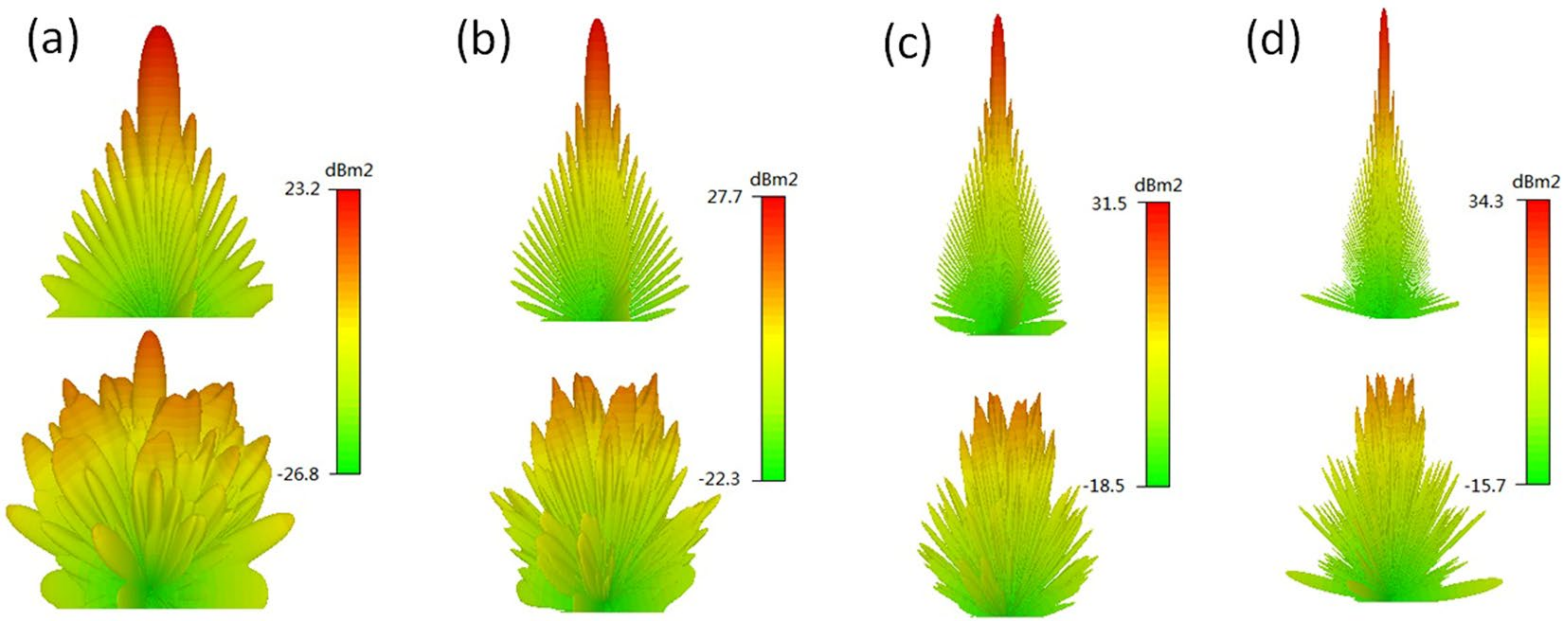
The bi-static RCSs of PEC ground plane and metasurface at four frequencies for normal incidence. (**a**) 6 GHz. (**b**) 10 GHz. (**c**) 16 GHz. (**d**) 22 GHz.

RCS can be divided into two types: monostatic RCS and bi-static RCS. Bi-static radar is the name given to a radar system which comprises a transmitter and receiver which are separated by a distance that is comparable to the expected target distance. Conversely, a radar in which the transmitter and receiver are collocated is called a monostatic radar. Therefore, the monostatic RCS represents the RCS value only in one direction, the normal direction. It is not related to other angle factors. The simulated monostatic RCS reduction of the metasurface is shown in Fig. 8. Less than −9.6 dB RCS reduction is achieved from 6.0 GHz to 25.5 GHz and there are four dips in the curve corresponding to four frequencies with exact 180° phase difference. The optimal metasurface can produce diffuse scattering with numerous lobes, leading to a great reduction of bi-static RCS. According to the Eq. (3), the bi-static RCS is related to two angle factors: *θ* and *φ*. The three-dimensional bi-static RCS patterns of the metasurface under normal incidence at 6 GHz, 10 GHz, 16 GHz and 22 GHz are depicted in Fig. 9, where *θ* ranges from 0° to 90° and *φ* ranges from 0° to 360°. Obviously, the bi-static RCSs are dramatically suppressed compared to that of the PEC ground plane.

As for the maximum or minimum value of RCS at a certain frequency, there are two methods usually used. One approach is to search the maximum value of RCS directly from the simulated RCS results in CST Microwave Studio. Another way is to output the bi-static RCS result from CST Microwave Studio and then select the maximum or minimum value by computer programming with MATLAB.

**Figure 10:**
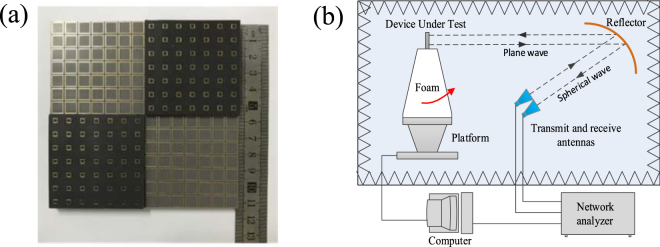
The fabricated metamaterial tile and the measurement setup. (**a**) The sample. (**b**) The schematic view of compact range system for the monostatic RCS measurement.

**Figure 11:**
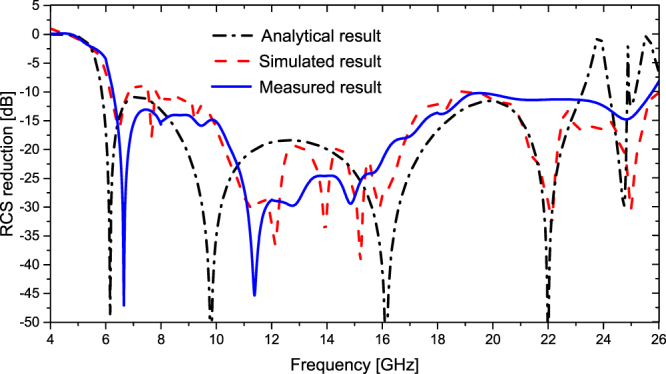
The analytical, simulated and measured RCS reductions of the metamaterial tile for normal incidence.

Measured results. To further study the predicted scattering characteristics, a metamaterial tile with the dimension of 112 mm × 122 mm is fabricated and measured. There are some reasons to adopt the sample with the dimension of 112 mm × 112 mm. First of all, the metamaterial tile with dimension of 112 mm × 112 mm is an uneven-layered coding element. A single metamaterial tile has realized the excellent monostatic RCS reduction. Multiple metamaterial tiles can be used to constitute lager experimental sample by optimizing the arrangement of the layout, which aims at reducing the bi-static RCS rather than the monostatic RCS. Furthermore, a high-precision RCS measurement is conducted using the compact antenna test range (CATR) system of the Science and Technology on Electromagnetic Scattering Laboratory in Beijing, China. The measurement accuracy is in line with the military standard, so the measured results are precise enough. Finally, taking the cost into account, the sample with the dimension of 112 mm × 112 mm is fabricated in the measurement. The dielectric substrate is PTFE woven glass substrate (Model: F4B-2, Wangling Insulating Materials, Taizhou, China) with a dielectric constant *ε*_*r*_ = 2.65 (loss tangent tan*δ* = 0.001). The metal patches and ground are 0.035 mm-thick copper layers. The sample is depicted in Fig. 10(a). The measurement setup of compact range system is shown in Fig. 10(b). Two identical horn antennas are utilized as transmitting and receiving devices, respectively. The spherical waves emitted by the horn antenna are reflected by the parabolic metal reflector and become plane waves. Short test distance between metamaterial tile sample and reflector is easy to meet the far field conditions. Four pairs of standard linearly polarized horn antennas are used to cover four frequency bands of 4–8 GHz, 8–12 GHz, 12–18 GHz and 18–26.5 GHz, respectively. The RCS of an equal-sized metallic surface is also measured as reference. The measured RCS of the metamaterial tile normalized to the equal-sized metallic surface is illustrated in Fig. 11. Less than −10 dB RCS reduction is achieved over an ultra-wide frequency band of 6.2–25.7 GHz. The analytical and simulated results are also depicted in Fig. 11. It is noted that the measured result coincides with the analytical and simulated results. Some deviation between them is attributed to the fabrication and measurement errors. Table 1 presents a comparison between our results and previous researches. It is found that the proposed metamaterial tile shows a great benefit in extending the bandwidth of RCS reduction. The ratio bandwidth reaches 4.15:1, and the relative bandwidth is up to 122.3%. Overall, the excellent performance of the proposed metamaterial tile has been confirmed.

**Table 1:** Comparison of Our Work and Previous Researches.

Article	RCSR (dB)	OFB (GHz)	BW (%)	RBW (${f}_{H}\,/{f}_{L}$)
^16^	10	14.5–21.8	41	1.50
^18^	10	9.3–15.5	50	1.67
^19^	10	9.40–23.28	85	2.48
^20^	10	10.5–18	52.63	1.71
^21^	10	3.94–7.40	61	1.88
^22^	10	7–12	52.63	1.71
^24^	10	6.1–17.8	98	2.92
^25^	10	7.5–15	66.67	2.00
^26^	10	7.9–20.8	89.9	2.63
^27^	10	17–42	84.75	2.47
This Work	10	6.2–25.7	122.3	4.15

RCSR: RCS reduction.

OFB: Operating frequency band.

BW: The relative bandwidth.

RBW: The ratio bandwidth.

## Conclusion

In this paper, the uneven-layered coding metamaterial tile is designed, fabricated and measured for ultra-wideband diffuse scattering and RCS reduction. The proposed metamaterial tile consists of two kinds of square ring unit cells with different layer thickness. The analysis and simulation results are consistent with the measurement results. The proposed metamaterial tile can achieve −10 dB RCS reduction over an ultra-wide frequency band from 6.2 to 25.7 GHz with a ratio bandwidth of 4.15:1. The metamaterial tile with the small size has the scattering pattern of four lobes deviating from normal direction. The metamaterial tile and its 90-degree rotation can be encoded as ‘0’ and ‘1’ elements, respectively. Both encoding elements can be used to cover objects, and diffuse scattering can be realized by optimizing the phase distribution, leading to low monostatic and bi-static RCSs simultaneously. This work is very helpful in the stealth and other microwave applications.
